# Exosomes in Pathogenesis, Diagnosis, and Treatment of Hepatocellular Carcinoma

**DOI:** 10.3389/fonc.2022.793432

**Published:** 2022-01-27

**Authors:** Shuang Li, Limin Chen

**Affiliations:** ^1^Institute of Basic Medicine, Shanghai University of Medicine and Health Sciences, Shanghai, China; ^2^Provincial Key Laboratory for Transfusion-Transmitted Infectious Diseases, Institute of Blood Transfusion, Chinese Academy of Medical Sciences and Peking Union Medical College, Chengdu, China; ^3^The Joint Laboratory on Transfusion-Transmitted Diseases (TTD) Between Institute of Blood Transfusion, Chinese Academy of Medical Sciences and Nanning Blood Center, Nanning Blood Center, Nanning, China

**Keywords:** exosomes, hepatocellular carcinoma, angiogenesis, metastasis, drug resistance, biomarker

## Abstract

Exosomes are extracellular vesicles with a diameter of 30-150 nm that are released by most types of cells and have been confirmed to be involved in many physical and pathological processes, especially in cell to cell communication. Compared with other vesicles, exosomes have a unique double-layer saclike structure that allows them to be present stably in various body fluids, including blood, cerebrospinal fluid, urine, saliva, and serous cavity effusion. The cargoes of exosomes reflect the characteristics of host cells. Due to the nature of hepatocellular carcinoma (HCC) cells, heterogeneity in the bioactive substances usually exist in exosomes. In addition, exosomes can efficiently deliver cargoes to the target cells to exert pathological functions, playing important role in tumor occurrence, development, metastasis, immune regulation, and drug resistance. Previous studies have been shown that exosomes have wide applications in diagnosis and treatment of HCC. In this review, we discuss these recent findings and highlight the significant roles of exosomes in HCC, focusing on the effect and underlying mechanisms of exosomes to regulate HCC progression and the potential clinical value of exosomes as biomarkers and therapeutic targets.

## Introduction

Hepatocellular carcinoma (HCC) is one of the most common malignant tumors worldwide, and many risk factors have been confirmed to be associated with HCC. These risk factors include but not limited to chronic HBV infection, chronic HCV infection, alcohol intake, non-alcoholic or metabolic fatty liver disease, autoimmune liver disease, genetic metabolic liver disease, aflatoxin exposure and type II diabetes (1, 2). Different approaches have been used to treat patients with HCC, such as surgery, liver transplant, ablation therapy, embolization therapy, targeted therapy, immunotherapy and radiation therapy (3–5). Despite extensive advances in treatment regimens, HCC shows a poor prognosis and high risk of recurrence leading it to be the fifth most lethal malignancy and the second cause of cancer-related deaths worldwide. Therefore, it is of great importance to understand the host factors involved in HCC progression, and to clarify the underlying mechanisms of HCC occurence and development in order to explore novel noninvasive biomarkers that can be used to facilitate early diagnosis, prognosis prediction and precision treatment for patients with HCC.

As one of the newly identified candidates for tumor biomarkers, exosomes are 30-150nm small vesicles with double-layer saclike structure. Various studies have demonstrated that exosomes can encapsulate components such as lipids, proteins and nucleic acids from host cells, and the exosomal membrane helps to protect these cargoes from enzymatic degradation. In addition, exosomes have other attractive features, such as low immunogenicity, high biocompatibility, and the ability to overcome biological barriers (6). Another catching feature of exosomes is that they exist widely and are stable in most body fluids such as in serum, plasma, lymph, saliva, urine, tears, sweat, semen, cerebrospinal fluid, and breast milk. As such, exosomes, together with circulating tumor DNA (ctDNA) and circulating tumor cells (CTCs), constitute the corner stones for liquid biopsy (7, 8). Contents (cargoes) encapsulated in the exosomes usually change with physiological and pathological conditions of the host cells. Numerous evidence indicates that exosomes exert intercellular communication *via* transporting these encapsulated intracellular components into the recipient cells to regulate a diverse range of pathological processes in cancers (9–13). Exosomes are involved in many processes of HCC, including tumor survival, growth, angiogenesis, invasion and metastasis. Exosomes also play a special role in the process of HHC by constructing a microenvironment suitable for HCC growth, such as providing energy, modulating signal pathways. Exosomes induce angiogenesis by changing the biological characteristics of endothelial cells and directly regulating angiogenic factors. In addition, exosomes may guide HCC metastasis and invasion through epithelial-mesenchymal transformation, extracellular matrix degradation and vascular leakage (14). In this review, we first summarize the major roles of exosomes played in HCC and then put forward views on how to make use of these recent progresses for future therapeutic applications. Due to the rapid growth, invasive and insidious onset of HCC, many patients have been diagnosed at an advanced stage which reduced the therapeutic effect. Therefore, effective early detection and diagnosis methods are of importance to improve the treatment and prognosis for HCC patients. Exosomes play a complex and important role in the occurrence, development, metastasis and recurrence of HCC. The detection and analysis of exosomes in body fluids of HCC patients can settle basic references for early diagnosis, treatment effect evaluation and prognosis of HCC. With more and more clinical data and sequencing results, the database is more perfect. At present, a large number of studies showed that the changes of some specific molecules in exosomes can be used as potential biomarkers for early diagnosis and prognosis evaluation for HCC patients. However, a single biomarker may not accurately reflect the occurrence and development of HCC, so it may be more inclined to use a combination of multiple exosomal markers. By summarizing these existing markers that may be helpful for the diagnosis of HCC, it may help to form a detection panel for HCC and improve the diagnostic accuracy.

## HCC-Derived Exosomes and Their Function

**Figure 1:**
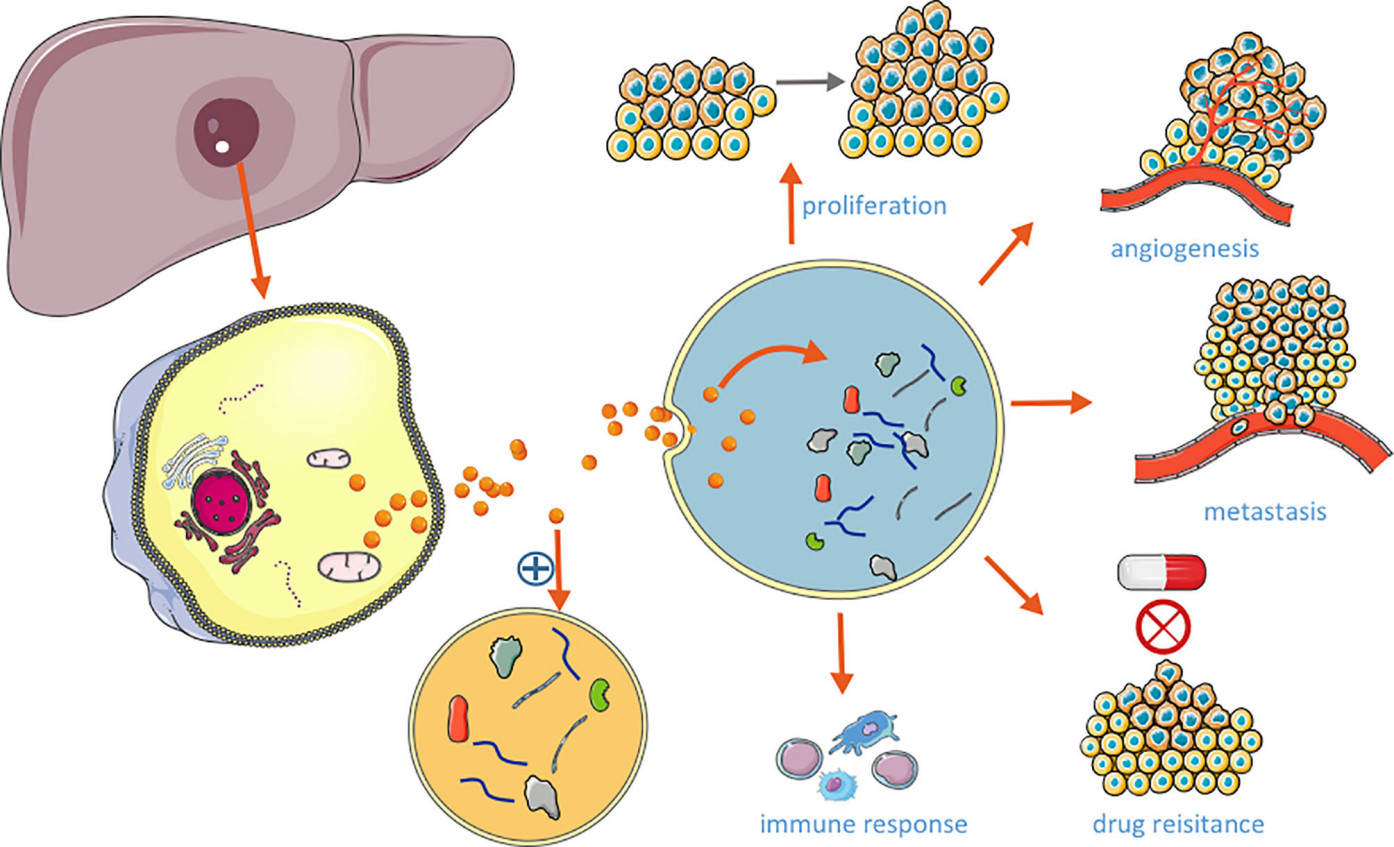
Role of HCC-derived exosomes in HCC.

There are some controversies around the nomenclature and classification of extracellular vesicles. According to the guidelines published by the international society for extracellular vehicles (ISEV) in 2018, extracellular vesicles can be classified according to their physical and chemical properties, such as the size and density of vesicles, and the specific proteins expressed by vesicles, which can be divided into three categories: exosomes, microvesicles and apoptotic bodies (15). Exosomes are small vesicles with a diameter of 30-150 nm formed through the fusion of multiple vesicles and cell membrane, and they can express CD63, CD81, CD9, TSG101, Alix and other markers (16). The amount of exosomes derived from HCC is associated with the tumor size, progression, and stage of the disease, and the expression levels of exosomal cargoes could reflect the status of the releasing cell. Emerging evidence has shown that the exosomal cargos enter the target cells, could trigger a cascade of signaling in recipient cells, facilitating tumorigenesis, angiogenesis, tumor growth and metastasis (**Figure 1**). Exosomes derived from HCC cells carry many functional molecules to exert their function in three ways: (1) fusion directly with the target cell membrane; (2) the binding between exosomal ligands and receptors of target cells; (3) endocytosis of the exosomes by target cells (17). These make the analyze of exosomal cargoes as a promising and noninvasive method to better determine the biology of HCC, and explore the directly targets of delivery cargoes as potential therapeutic for HCC.

## Exosomes in the Pathogenesis and Progression of HCC

The development and progression of HCC is determined by not only the malignant potential of the tumor cell itself but also the signals from its microenvironment (18). Tumor microenvironment refers to the internal environment in which tumor cells replicate and live. It includes not only the tumor cells themselves but also the fibroblasts, inflammatory cells, endothelial cells and other cellular components, as well as extracellular matrix, growth factors, inflammatory cytokines, proteolytic enzymes and their inhibitors (19). Exosomes have been shown to play a key role in the crosstalk between tumor cells and their surrounding microenvironment, and the transfer of exosomal proteins and nucleic acids could induce phenotypic changes in target cells (20, 21). Accumulating evidence suggests that HCC-derived exosomes facilitate tumor development and progression by generating a favorable milieu through angiogenesis, immune suppression, or even drug resistance. Epithelial mesenchymal transition (EMT) refers to the morphological transformation of epithelial cells into mesenchymal cells, during the process, epithelial cells loosen their attachments to neighboring cells, lose apico-basal polarity, become elongated and display increased motility, and accompanied by dissolution of adherens junction proteins and disruption of tight junctions, resulting in dissociation of epithelial cells. Such as the expressions of epithelial markers E-cadherin and Zonula occluden-1 are down-regulated, and the expressions of mesenchymal markers Vimentin and N-cadherin are up-regulated. In HCC, the EMT state is associated with tumor initiation, invasion, metastasis, and resistance to therapy. A variety of signal pathways are involved in the EMT process, such as Wnt, TGF- β (transforming growth factor- β), c-Met and Notch-1 signaling pathways, etc. The Wnt/β-Catenin pathway appears to drive the EMT of HCC, to effectively inhibiting various targets in the Wnt signaling pathway can reduce or reverse the occurrence of tumor EMT. During EMT, the anti-apoptotic effecter NK-κB activated *via* the Notch-1 pathway and TGF-β may regulate tumor microenvironment through TGF-β/SMAD signaling. Moreover, TGF-β And Notch signal may cooperate with other signal pathways to induce tumor EMT (22, 23). In addition to various signal pathways, some cytokines, transcription factors, matrix metalloproteinases and viral oncogenes are also involved in the process of tumor EMT, and the newly studied noncoding RNAs also play a very important role in tumor EMT and its signal pathways (24). Through autocrine and paracrine, tumor derived exosomes provide signals to the microenvironment to activate the EMT progress so that tumor cells may invade the surrounding tissues and enter the circulation.

### Exosmal RNAs in HCC Development and Progression

Exosomal RNAs have unique expression profiles reflecting the characteristics of tumors, and their role in tumor progression and metastasis is gradually emerging (25–27). Recent studies demonstrated that the imbalance of non-coding RNA expression is involved in many pathological processes of HCC development and progression (28). Non-coding RNA (ncRNA) refers to RNA that can be transcribed from the genome, but can not be translated into proteins. With the in depth research, more and more evidence supports that ncRNA is not useless “garbage”, in stead, many lncRNA (long non-coding RNA), miRNA (microRNA), circRNA (circular RNA) can participate in a variety of biological processes, playing important biological functions at the RNA level (29, 30). Many studies have found that ncRNA plays a regulatory role in the occurrence and development of HCC (31, 32). Most importantly, ncRNAs can be wrapped into exosomes to be transferred from cell to cell.

Tumor and its microenvironment are not only interdependent and promoting each other, but also they are antagonistic and struggling with each other. In the process of interaction between tumor cells and their microenvironment, exosomes function as a vector for intercellular communication. In the early-stage of HCC the acidic environmental is correlated with poor prognosis. Further study revealed the exosomes derived from HCC cells cultured in the acidic medium promoted proliferation and metastasis of recipient HCC cells. Moreover, exosomal miR-21 and miR-10b as the most important functional miRNAs significantly stimulated HCC cell proliferation, migration, and invasion both *in vivo* and *in vitro* (33). HCC cells derived exosomes overexpress circRNA Cdr1as, the circRNA Cdr1as could serve as a ceRNA to sponge miR-1270 and promote the expression of AFP which is the target gene of miR-1270, thereafter accelerating proliferation and migratory abilities of HCC cells (34). The oncogene lncRNA FAL1 was up-regulated in HCC tissues and functioned as ceRNA to bind miR-1236 to accelerate cell proliferation and metastasis. Moreover, lncRNA FAL1 could be transferred by exosomes to surrounding HCC cells to increase their abilities of cell proliferation and migration. This makes exosomal lncRNA FAL1 may be a novel diagnostic biomarker or a novel target for the treatment of HCC in the future (35). Hypoxia plays a pivotal role in the progression of many tumor types. The growth rate of blood vessels is slower than that of HCC cells, resulting in the tumor tissues and cells hypoxia, and the hypoxic microenvironment further activates angiogenic factors such as vascular endothelial growth factor (VEGF) and initiates abnormal angiogenesis. The abnormal structure and function of new vessels aggravate the tumor hypoxic microenvironment and promote the development and metastasis of HCC (36). A previous study found that hypoxic conditions enhanced the secretion of exosomes by HCC cells, and these exosomes enhanced proliferation, migration, invasion, and epithelial-to-mesenchymal transition in normoxic HCC cells. Remarkably enhanced miR-155 expression level was observed in the exosomes derived from human liver cancer cell lines (PLC/PRF/5 and HuH7) under hypoxia and increased tube formation was noted from human umbilical vein endothelial cells (HUVECs) in the presence of these exosomes. MiR-155 was a major player in this pathogenesis because the increased tube formation effect was attenuated if miR-155 in PLC/PRF/5 or HuH7 cells were knocked down (37). Moreover, researchers found that exosomal miRNA-21 and miR-155 from HCC cells could directly target PTEN, leading to the activation of PDK1/AKT signaling in hepatic stellate cells (HSCs), which promoted cancer progression by secreting angiogenic cytokines, including VEGF, MMP2, MMP9, bFGF and TGF-β (38). In addition, exosomal miR-1273f was increased under hypoxic conditions and miR-1273f activated the Wnt/β-catenin signaling pathway leading to enhanced malignant phenotype (39). MicroRNA-23a/b (miR-23a/b) was significantly upregulated in exosomes of HCC patients with a high body fat ratio than low body fat ratio. The exosomal miR-23a/b promotes proliferation of HCC cells by directly targeting the tumor suppressor VHL. The VHL protein directly mediates the ubiquitylation and proteasomal degradation of HIF-1α which is the key transcription factor of oxygen homeostasis regulation. The results demonstrated that the new axis of miR-23/VHL/HIF-1α may play a key role in HCC development with high body fat ratio (40). These results suggest that under hypoxia condition, HCC-derived exosomes were able to regulate the pathogenesis of HCC *via* transporting some functions molecules, such as miRNAs. It has been observed that exosomal circRNA-deubiquitination (circ-DB) is upregulated in HCC patients with higher BMI (body fat ratio). Moreover, *in vitro* and *in vivo* studies showed that exo-circ-DB promotes HCC growth and reduces DNA damage *via* the suppression of miR-34a and the activation of deubiquitination-related USP7. This stimulating effect of exosomes from adipose on HCC cells can be reversed by knockdown of circ-DB (41). These results suggest that the molecular composition of exosomes released by cells under different stress microenvironments varies greatly and some specific functional molecules contained in exosomes mediate the communication between tumor and tumor microenvironment, affecting disease pathogenesis and progression.

Blood vessels not only provide oxygen and nutrition for tumor growth but also played an important role in tumor metastasis (42). Tumor vascular endothelial cells are regulated by a variety of cytokines secreted by tumor cells and exosomes are largely involved in modulating the interactions between HCC and endothelial cells through transfering some biological molecules such as miRNAs, lncRNAs and circRNAs. It has been shown that miR-210 in HCC-secreted exosomes stimulates tube formation in endothelial cells by targeting SMAD4 (SMAD Family Member 4) and STAT6 (signal transducer and activator of transcription 6) (43). Exosomal miR-378b from HepG2 cells binds with transforming growth factor β receptor III (TGFBR3) in HUVECs to down-regulate TGFBR3 expression which is negatively associated with HCC metastasis and angiogenesis. As such, upregulated exosomal miR-378b could promote HCC metastasis and angiogenesis (44). In another study, miR-1290 was over-expressed in exosomes derived from HCC patient serum. As the sponge of SEMK1 (suppressor of mitogen-activated protein kinase kinase 1), the exosomal miR-1290 increases VEGFR2 (vascular endothelial growth factor receptor 2) phosphorylation in human endothelial cells to promote the HCC angiogenic ability (45). miR-638 has been shown to inhibit cell proliferation of HCC by decreasing viability and colony formation and inducing apoptosis and cell cycle arrest at G1 phase. In addition, exosomal miR-638 suppressed HUVEC proliferation, migration and invasion *via* decreasing SP1 (46). In agreement with this, the proliferation of Huh7 and SMCC7721 HCC cells were significantly inhibited when miR‐638 was over‐expressed in these cells (47).

Upregulated in both serum from HCC patients and HCC cells, the exosomal lincRNA LINC00161 was directly bound to miR-590-3p to upregulate its downstream target gene ROCK2 (Rho-associated protein kinase 2), stimulating cell proliferation, migration and angiogenesis of HUVECs (48). The linc-FAM138B level in exosomes from cancer cells of HCC patients was lower than that in exosomes from normal cells. As a sponge for miR-765, linc-FAM138B promoted the growth of cancerous hepatocytes by decreasing miR-765 expression (49). CircRNA-100338 was highly expressed in highly metastatic HCC cells and their secreted exosomes. Exosomal circRNA-100338 can regulate HUVECs’ angiogenesis *via* increasing cell proliferation, angiogenesis, permeability, and vasculogenic mimicry formation ability (50). Exosomes isolated from a highly metastatic HCC cell line (LM3) enhanced the cell migration and invasion potential of HepG2 (non-metastatic cell line) and 97 L (low-metastatic cell line). CircPTGR1 is highly expressed in exosomes derived from the metastatic HCC cell line LM3. By targeting miR449a, circPTGR1 stimulates mesenchymal-epithelial transition factor expression which can promote migration and metastasis in HCC (51). Taken all these data together, exosomes, especially those biologiocally-active molecules wrapped into the exosomes have potential application in predicting HCC progression and prognosis.

The tumor microenvironment is composed of tumor cells, vascular endothelial cells, fibroblasts, antigen-presenting cells, extracellular matrix, growth factors and inflammatory cytokines. Different exosomes play a role in cell communication by carrying different signal molecules and participate not only in the occurrence, metastasis and angiogenesis, but also modulate anti-tumor immune responses. Immune cells are an important part of the tumor matrix and play an indispensable role in tumor growth, differentiation, and immune escape. Immunosuppressive cells can also promote the secretion of VEGF and other cytokines and chemokines (52). Exosomes are now considered as important mediators of host anti-tumor immune response as well as tumor cell immune escape. Recently, emerging evidence suggestes tumor associated macrophages (TAMs) is involved in tumor progression (53). Accumulating evidence indicated that macrophages-derived exosomes regulate HCC progression. MiR-326 was decreased in exosomes isolated from HCC cells but enriched in those from M1 macrophages. M1 Macrophage-derived exosomes deliver miR-326 to HCC to reduce cell proliferation, colony formation, migration, invasion, and CD206 and NF-κB (nuclear factor kappa-B) expression and to promote apoptosis (54). Another study demonstrates that exosomal miR-125a/b can target the HCC stem cells marker CD90 and suppress cell proliferation and stem cell properties (55). Furthermore, exosomal miR-146a-5p plays a key role in macrophage M2 polarization by activating NF-κB signaling. In addition to miRNAs, other non-coding RNAs such as cirRNAs wrapped in exosomes are also involved in the modualtion of macrophage activation and polarization to affect HCC progression. For example, hsa_circ_0074854 expression was upregulated in both HCC tissues and HCC cell lines and this circ-RNA can be transferred to macrophages *via* exosomes. Study has shown that knockdown the expression of hsa_circ_0074854 in HCC cells decreased the exosomal hsa_circ_0074854 entering macrophages and suppressed macrophage M2 polarization, and in turn inhibited migration and invasion of HCC cells both *in vitro* and *in vivo* (56). LncRNA TUC339 was enriched in HCC-derived exosomes and can be transferred to neighbor macrophages and was positively associated with M(IL-4) macrophages polarization (57). Interestingly, circUHRF1 in exosomes from HCC patient sera is not only associated with a decreased NK cell proportion but also associated with the impaired IFN-γ and TNF-α secretion of NKs by up-regulating the expression of TIM-3 *via* degradation of miR-449c-5p (58). Moreover, the macrophages educated with HCC-derived exosomes inhibited T cell response by upregulating the expression of inhibitory receptors such as PD-1 (programmed death-1) and CTLA-4 (cytotoxic T-lymphocyte associated protein 4) (59). These results demonstrated that exosomes are important in alerting the host immune and inflammatory cells to sense the presence of cancer cells. Moreover, exosomes secreted from tumor microenvironment can educate surrounding cells to create a more favorable microenvironment for HCC progression. Although exosomal cargos are of different origins and have various mechanisms of action, exosomes play an increasing role in the regulation of immune response to tumors.

### Exosmal Proteins in HCC Development and Progression

It has been reported that exosomes derived from hepatoma cells can promote the migration and invasion of recipient cells. Moreover, highly invasive hepatoma-cells (MHCC97H)-derived exosomes have stronger promoting effects than those from low-invasive hepatoma cells (MHCC97L) and normal liver cells (LO2) exosomes. Further studies indicated that MHCC97H and MHCC97L-derived exosomes induce the decrease of E-cadherin expression and the increase of Vimentin expression to promote EMT in recipient cells *via* TGF-β/Smad signaling pathway (60). Human cells respond to internal and external stimuli by altering the level and activity of their proteins, so exosomal proteins may change either qualitatively or quantitatively to exert functional effects in the pathogenesis and progression of HCC. Proteomics analysis revealed that compared with exosomes from non-motile HCC cell line (Hep3B), 469 proteins were differentially expressed in exosomes from the motile HCC cell line 97H and the expression levels of another 443 exosomal proteins were significantly changed in another motile HCC cell line LM3. These differentially-expressed exosomal proteins were enriched in the sugar metabolism-centric canonical pathways, therefore these exosomal sugar metabolism proteins may be new biomarkers for more motile liver cancer cells (61). Proteins are directly involved in almost every biological process, so comprehensive analysis of these differentially-expressed proteins in HCC cells and HCC-derived exosomes may shed light to explain how these proteins interact and cooperate to affect the development and progression of HCC. Vasorin is a type I transmembrane protein that plays important role in tumor development and vasculogenesis. Previous study demonstrated that the HepG2-derived exosomal Vasorin transferred to human umbilical vein endothelial cells (HUVECs) *via* proteoglycans mediated endocytosis could promote migration of recipient HUVECs (62). Results from this study indicated that vasorin could be one of the key mediators of communication between tumor cells and endothelial cells. C-Type Lectin Domain Family 3 Member B (CLEC3B) is a transmembrane Ca^2+^-binding protein and down-regulated CLEC3B in HCC indicated a poor prognosis. The CLEC3B can be wrapped inside exosomes and the downregulated exosomal CLEC3B suppressed vascular endothelial growth factors (VEGFs) secretion in both HCC cells and endothelial cells *via* AMPK (adenosine monophosphate-activated kinase) signal pathway, and eventually inhibited angiogenesis (63). HCC is a typical hypervascular solid tumor that requires neoangiogenesis for growth. Angiopoietin-2 (ANGPT2) has been shown to be able to destroy vascular stability to promote cancer angiogenesis and the level of ANGPT2 is closely related to the development and prognosis of HCC, the HCC-derived exosomal ANGPT2 increased the tubule formation, migration and proliferation of HUVECs leading to enhanced angiogenesis of HUVECs *in vitro* (64). Lysyl oxidase-like 4 (LOXL4) was upregulated in HCC and predicted a poor prognosis. HCC-derived exosomes transferred LOXL4 not only between HCC cells to promote cell migration by activating the FAK/Src pathway but also to HUVECs through a paracrine mechanism to stimulate angiogenesis (65). EIF3C (eukaryotic translation initiation factor 3 subunit C) is also upregulated during HCC tumor progression and is associated with poor patient survival. Upregulated EIF3C expression in HCC cells (PLC5) increased the release of exosomes and enhanced angiogenesis *in vitro* and *in vivo*. It has been shown that EIF3C upregulated the expression level of S100 calcium-binding protein A11(S100A11), which plays critical roles in cancer progression and angiogenesis in PLC5 cells, and treatment with exosome inhibitor GW4869 or suppression of S100A11 expression could abolish the EIF3C-mediated HCC angiogenesis (66). These data indicated EIF3C mediated tumor progression *via* increasing release of oncogenic exosomes to potentiate angiogenesis and tumorigenesis in tumor microenvironment. S100 calcium-binding protein A4 (S100A4) plays an important role in tumor metastasis by regulating adhesion, extracellular matrix remodeling, and cellular motility. Exosomes derived from highly metastatic HCC cells (HMH) can transfer abundant S100A4 to low metastatic HCC cells (LMH) to enhance the stemness and metastatic potential, and this effect is mediated by induced expression of osteopontin *via* STAT3 phosphorylation (67). Alpha-enolase (ENO1) is frequently upregulated in HCC cells or tissues and can be transferred between cells *via* exosomes. The exosomal ENO1 promoted cellular malignant transformation and metastasis of HCC cells by activating the FAK/Src-p38MAPK pathway and upregulating integrin α6β4 expression which is closely related to tumor growth and metastasis (68). In addition to cancer cells, proteins wrapped in the exosomes may also affect the function of other cell types in the tumor microenvironment. Impaired activation, proliferation and anti-tumor functions of tumor-infiltrating T lymphocytes (TILs) have been observed after treatment with 14-3-3ζ-containing exosomes derived from HCC cells (69). These findings implicated that some exosomal proteins may be used as novel biomarkers for the clinical evaluation of HCC progression and metastasis. In the process of exosome production, exosomes are filled with biological proteins, which are transferred from donor cells to recipient cells. These characteristics contribute to the role of exosomes in intercellular communication. The size and content of exosomes vary greatly, and their biological function and targets are also different. Therefore, exosomes have attracted much attention as important carriers of specific signals that may play important roles in the regulation of HCC progression, metastasis, angiogenesis and immune response (**Table 1**).

**Table 1:** Primary functions of non-coding RNAs and proteins in exosomes from HCC.

Function	Cargo	Effect	Ref.
Proliferation	miR-21	Promote	(33)
	miR-10b	Promote	(33)
	circRNA Cdr1as	Promote	(34)
	miR-23a/b	Promote	(40)
	circ-DB	Promote	(41)
	miR-638	Repress	(46)
	LINC00161	Promote	(48)
	linc-FAM138B	Inhibit	(49)
	circRNA-100338	Promote	(50)
	miR-326	Repress	(54)
	miR-125a/b	Inhibit	(55)
Tube formation	miR-155	Promote	(37)
	miR-21	Promote	(38)
	miR-210	Promote	(43)
	miR-378b	Promote	(44)
	miR-1290	Promote	(45)
	circRNA-100338	Promote	(50)
	miR-638	Inhibit	(46)
	LINC00161	Promote	(48)
	Vasorin	Promote	(62)
	CLEC3B	Inhibit	(63)
	ANGPT2	Promote	(64)
	LOXL4	Promote	(65)
	EIF3C	Promote	(66)
Metastasis	miR-1273f	Promote	(39)
	miR-378b	Promote	(44)
	LINC00161	Promote	(48)
	circPTGR1	Promote	(51)
	VASN	Promote	(63)
	S100A4	Promote	(67)
	ENO1	Promote	(68)
Immune response	hsa_circ_0074854	Suppress macrophage M2 polarzation	(56)
	LncRNA TUC339	Positive associated with M(IL-4) macropaghe polarzation	(57)
	circUHRF1	Inhibit NK cell function	(58)
	miR-146a-5p	Promote macrophage M2 polarzation	(59)
	14-3-3ζ	Inhibit tumor infiltrating T lymphocytes activation, proliferation and anti-tumor functions	(69)
Drug resistance	lincRNA-VLDLR	Resistance to sorafenib, camptothecin and doxorubicin	(70)
	lincRNA-ROR	Sorafenib resistance	(71)
	miR-744	Sorafenib resistance	(72)
	miR-32-5p	Induce multidrug resistance	(73)
	circRNA-SORE	Spread sorafenib resistance	(74)

### Exosomes in Drug Resistance of HCC

HCC is the most common primary liver cancer and drug resistance is the predominant obstacle for the treatment of HCC. With the development of gene sequencing technology, the gene mutation map of HCC is more clear. Some major mutant genes and corresponding signal pathways related to hepatocarcinogenesis have been found, such as Wnt/β- Catenin, chromatin remodeling, p53/cell cycle, MAPK, BRAF, mTOR, etc (75). Among the main signal pathways, the MAPK pathway and BRAF signaling play a crucial role in the regulation of HCC cell proliferation and survival. Tyrosine kinase inhibitors exert their activity against HCC cells inhibiting BRAF signaling. Sorafenib, a multi-target kinase inhibitor for the treatment of HCC, can directly inhibit tumor growth by blocking BRAF and MAPK signal pathways, previous studies have shown that sorafenib can effectively prolong the median overall survival of patients with advanced hepatocellular carcinoma (76, 77). However, many HCC patients will develop sorafenib resistance after 6 months treatment, due to several mutations HCC cells often presented an intrinsic and acquired resistance to tyrosine kinase inhibitors, and then progress to metastasis, which makes the next step of treatment very difficult. The mechanisms of drug resistance can be multifactorial. The genome of HCC cell can be reprogrammed to acquire resistance to treatment after exposure to chemotherapy or targeted therapy. In addition, some defense pathways for tumor cells, such as changes in cell cycle checkpoints or DNA damage repair mechanisms, can also cause tumor drug resistance (78). Interestingly, accumulatting evidence suggested that exosomes may also play an important role in drug resistance of patients with HCC. The exosomal lncRNA mediators, such as lincRNA-ROR, are involved in the modulation of hepatoma cellular responses to sorafenib. Moreover, lincRNA-VLDLR could be transferred by HCC-derived exosomes to mediate resistance to anti-cancer agents, such as sorafenib, camptothecin, and doxorubicin, in recipient cancer cells (70, 71). Exosomal miR-744 is downregulated in exosomes derived from HCC patient serum and HepG2 cells. It has been shown that suppressed miR-744 promotes HepG2 cell proliferation and inhibits the chemosensitivity of HepG2 cells to sorafenib *via* regulating PAX2 (paired box 2) expression (72). The PTEN/PI3K/Akt pathway contributes to chemoresistance in different types of cancers by regulating proliferation, apoptosis, angiogenesis and autophagy (79–81). The drug-resistant HCC cell line Bel/5-FU delivers miR-32-5p to the sensitive HCC cell line Bel7402 by exosomes to reduce PTEN expression,which in turn activates the PI3K/Akt pathway leading to multidrug resistance by modulating angiogenesis and epithelial-to-mesenchymal transition (73). Exosomes derived from HCC cells induced sorafenib resistance both *in vitro* and *in vivo* by activating the HGF/c-Met/Akt signaling pathway and inhibiting sorafenib-induced apoptosis. Moreover, exosomes derived from highly invasive tumor cells (MHCC-97H) had greater efficacy in stimulating HCC cell proliferation and in hibiting the chemotherapeutic effects of sorafenib than those derived from less invasive cells (MHCC-97 L) (82). CircRNA-SORE is upregulated in sorafenib-resistant HCC cells, and circRNA-SORE binds the master oncogenic protein YBX1 (Y box binding protein 1) to prevents its interaction with the E3 ubiquitin ligase to block YBX1 degradation. Moreover, the circRNA-SORE can be transported by exosomes among HCC cells to spread sorafenib resistance to other sensitive cells. As expected, silencing circRNA-SORE could substantially overcome the resistance to sorafenib (74). In the process of HCC occurrence and development, due to the influence of tumor cells and the microenvironment, there will be different degrees of heterogeneity in HCC cells, which may contribute to different sensitivity to chemotherapeutic drugs in the same tissue. And the inconsistency of chemosensitivity between tumor cells can be transferred between cells *via* exosomes to make sensitive cells to obtain drug resistance.

## Potential Applications of Exosomes in HCC Diagnosis and Treatment

### Exosomes as Biomarkers

Exosomes not only promote the malignant progression of HCC but also can be used as biomarkers for auxiliary diagnosis, efficacy evaluation, and prognosis monitoring in HCC. In recent years, exosomes as disease diagnosis biomarkers have gained a lot of attention and gradually become a hot research topic. Accumulating evidence indicated that exosomes and their cargoes are clearly related to the onset and development of HCC. In patients with early stage HCC, serum exosomal miR-21 and miR-10b levels were associated with tumor stages and they were independent prognostic biomarkers for disease-free survival (33). Data from 79 HCC patients demonstrated that circulating exosomal miRNA-21 and exosomal lncRNA-ATB were related to TNM (Tumour-Node-Metastasis) stage and portal vein thrombosis. In addition, both higher miRNA-21 and higher lncRNA-ATB were independent predictors of mortality and disease progression (83). The persistent high expression of exosomal circRNA-100338 in serum of HCC patients who underwent curative hepatectomy may be a biomarker of pulmonary metastasis and poor survival (50). LINC00161 was enriched in exosomes derived from serum of HCC patients (N=56), and high exosomal LINC00161 expression was associated with significantly poor survival (48). The circPTGR1 was upregulated in serum exosomes isolated from HCC patients and was associated with the clinical stage and prognosis of HCC, indicating their prognostic value in the clinical setting (51). A cohort study consisting of 40 patients with HCC found that higher expression level of exosomal miR-155 in preoperative plasma was significantly correlated with early recurrence (37). A study focused on viral HCC found that the exosomal miRNAs (miR-10b-5p, miR-21-5p, miR-221-3p, miR-18a, miR-221, miR-222 and miR-224) significantly upregulated in HCV and HBV associated HCC patients than normal/non-HCC group including chronic hepatitis and liver cirrhosis patients, and exosomal mir-223-3p, miR-101, miR-106b, miR-122 and miR-195 showed the opposite trend (84). All these studies strongly suggest the altered levels of some specific molecules wrapped in the exosomes can be used as potential biomarkers for the early diagnosis and prognosis evaluation of patients with HCC. Moreover, a single biomarker may not be sufficiently accurate for HCC diagnosis, and use of a combination of diffrent biomarkers from HCC-derived exosomes may be prefered.

**Figure 2:**
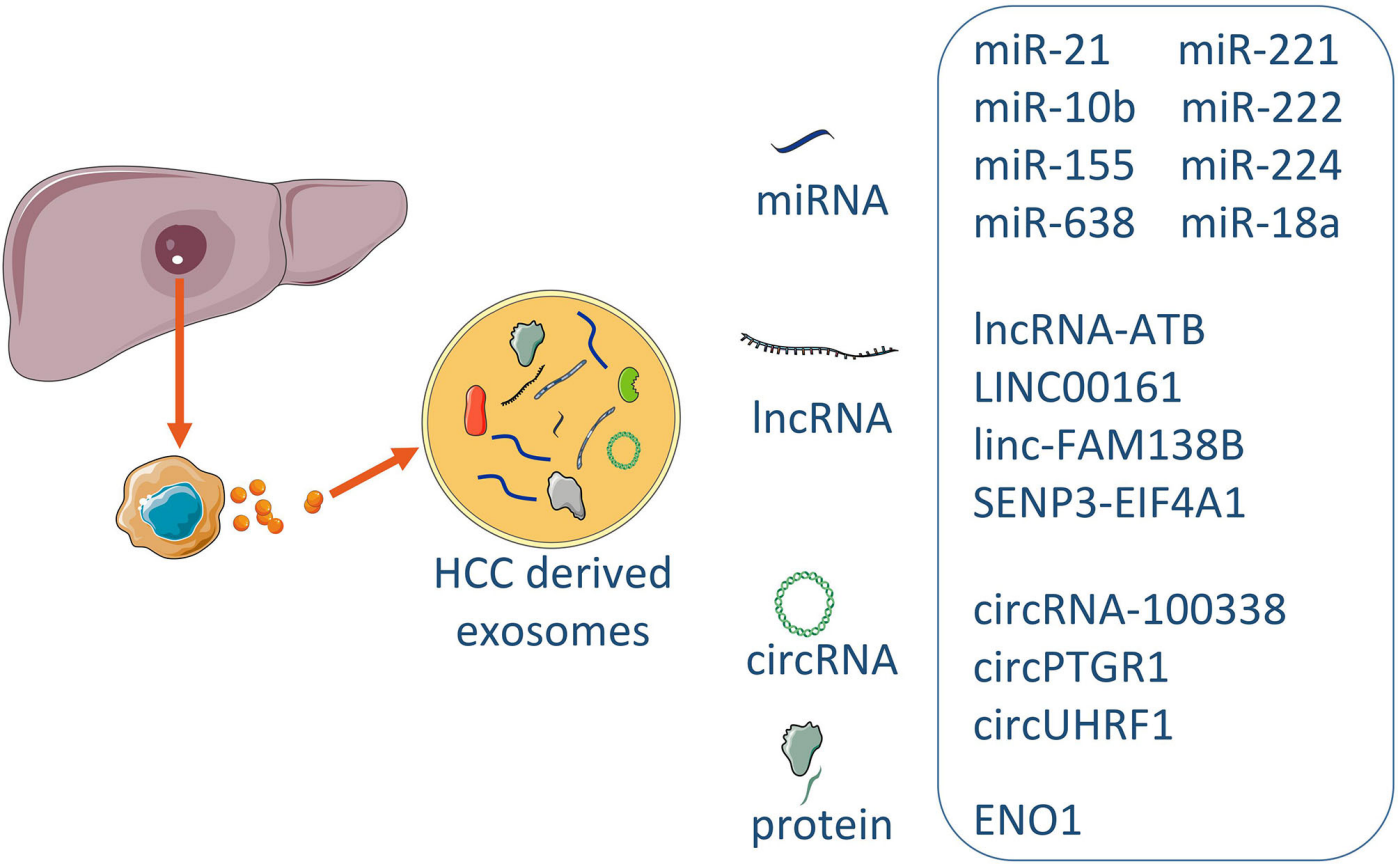
The exosomal biomarkers in HCC. Exosomes derived from HCC contained the different consist of cargoes of miRNAs, lncRNAs, circRNAs and proteins. Some specific molecules can be utilized for typical exosome biomarkers, and early prediction and prognosis of disease.

Plasma exosomal circUHRF1 levels were increased in HCC patients compared with healthy individuals. Interestingly, plasma exosomal circUHRF1 levels were significantly increased in patients with immunosuppression. These data suggested that plasma exosomal circUHRF1 was closely related to poor prognosis in patients with HCC (58). Clinical data showed that ENO1 expression was higher in metastatic lesions than that in the primary lesions and that ENO1 upregulation was significantly correlated with the TNM stage and tumor differentiation grade, and predicted a poor prognosis in patients with HCC. Moreover, the expression trend of ENO1 in exosomes was consistent with that in cells, although the exosomal ENO1 level cannot fully reflect the proliferative activity of cells, the expression level of exosomal ENO1 was positively correlated with cell migration and invasion capabilities (68). HCC patients with lower levels of serum exosomal miR‐638 had poorer overall survival than those with higher levels of exosomal miR‐638 in serum (47). In addition, a negative association of serum exosomal miR‐638 with tumor size, vascular infiltration, and TNM stage was observed in patients with HCC. Linc-FAM138B was reduced in both HCC tissues and cell lines and lower level expression of linc-FAM138B indicated a poorer prognosis in HCC patients. Although decreased expression of linc-FAM138B in exosomes of cancer cells from HCC patients was also observed but the correlation between the expression levels of exosomal linc-FAM138B and survival rate of patients with HCC needed to be further investigated (49). Long non-coding RNA SENP3-EIF4A1 stimulated apoptosis and weakened the invasion and migration abilities of HCC cells and modulated the expression of ZFP36 (the negative regulator for HCC migration and invasion) by competitively binding to miR-9-5p. Exosomal SENP3-EIF4A1 was significantly reduced in HCC patients and may exert as a biomarker for clinically detecting HCC (85). Exosomes contain proteins and nucleic acids similar to those of HCC cells. The structure of lipid membrane of exosomes can protect the contents wrapped in exosomes from being degraded, so the nucleic acids and proteins in the exosomes are relatively stable, making them good biomarker candidates (**Figure 2**).

### Exosomes as Vehicle for the Delivery of Therapeutic Agents

Exosomes can be modified with a variety of molecules, thereby acting as a vehicle for the delivery of therapeutic agents. Because of the easy degradation of RNAs or functional molecules by direct infusion, effective vehicle-mediated delivery may represent a new strategy for improving the efficacy of HCC chemotherapy. Exosomes can carry some specific functional molecules to increase the sensitivity to cancer drugs and reverse the drug resistance of HCC. Mesenchymal stem cells (MSC) can be harvested from several adult tissues and they can constitute a heterogeneous subset of stromal regenerative cells (86). Similar to exosomes in general, MSC-derived exosomes carry complex cargo, and are therefore well equipped to maintain homeostasis within the tissue and respond to external stimuli. Compared with MSC, the exosomes from MSC have no cell activity, and there is no risk of tumor formation (87). In addition, the immunogenicity of exosomes is lower than that of MSC due to the low content of membrane binding proteins (88). These characteristics make MSC-derived exosomes an ideal delivery system for cancer treatment (89). More and more studies have proved their safety and effectiveness against HCC. The GRP78 is overexpressed in sorafenib resistant cancer cells compared to sorafenib sensitive cancer cells and thus is a potential target for the treatment of HCC. BM-MSCs were modified to express siGRP78 which is able to target GRP78 to decrease the expression level of GRP78. Exosomes with siGRP78 (exo-siGRP78) were isolated from the cultured BM-MSCs (bone-marrow-derived mesenchymal stem cells). Combination therapy with exo-siGRP78 and sorafenib significantly inhibited the growth and invasion of the HCC cells *in vitro* as well as *in vivo* (90). The loss or downregulation of liver-specific miR-122 has been associated with HCC development and progression (91) and is closely related to poor prognosis and metastasis of HCC (92). Increasing evidence indicates that miR-122 can modulate the sensitivity of HCC cells to doxorubicin and sorafenib. Exosomes as biological vehicles for miRNA transfer do not elicit acute immune rejection and no risk of tumor formation. Data showed that miR-122-transfected adipose tissue-derived MSC (AMSCs) can effectively package miR-122 into secreted exosomes, which can mediate miR-122 transfer between AMSC and HCC cells, thereby rendering cancer cells sensitive to chemotherapeutic agents through alteration of miR-122 gene expression in HCC cells. Moreover, intra-tumor injection of highly expressed miR-122-exosomes significantly increased the antitumor effect of sorafenib on HCC *in vivo*. These findings suggest that the transfer of miR-122 *via* AMSC exosomes represents a novel strategy to enhance HCC chemosensitivity (93). In xenograft mice, overexpression of miR-638 reduced HCC growth *via* decreasing SP1 (specificity protein 1), indicating a potential clinical application of miR-638 in the treatment of HCC (46). MiR-199a-3p (miR-199a) has been shown to enhance the chemosensitivity of HCC. Transfected AMSCs with miR-199a lentivirus can secrete exosomes (AMSC-Exo-199a) containing a high level of miR-199a. AMSC-Exo-199a had the classic characteristics of exosomes and could effectively deliver miR-199a into HCC cells to enhance HCC sensitivity to doxorubic through ininhibiting the mTOR pathway. Moreover, i.v.-injected AMSC-Exo-199a could be successfully distributed to tumor tissue and markedly increase the effect of doxorubicin against HCC *in vivo* (94). Exosomes isolated from the human umbilical cord mesenchymal stem cell (HucMSC) contain miR-451a to be able to down-regulate the expression level of its target gene ADAM10 in Hep3B and SMMC-7721 cells. Decreased expression of ADAM10 reverts the paclitaxel resistance and inhibits cell cycle transition, proliferation, migration and invasion, and promotes apoptosis of HCC cells. These data demonstrated that HucMSC-derived exosomal miR-451a could provide a new strategy for HCC treatment by targeting ADAM10 (95).

Cancer-associated fibroblasts play a pivotal role in regulating tumor progression. Therefore, understanding how cancer-associated fibroblasts communicate with HCC is crucial for HCC therapy. A study found miR-320a is reduced in the exosomes of cancer-associated fibroblasts from HCC patients. *In vitro* and *in vivo* studies further revealed that miR-320a is an antitumor miRNA by binding to its direct downstream target PBX3 (pre-B cell leukemia 3) to suppress HCC proliferation, migration and metastasis. The miR-320a-PBX3 axis inhibited tumor progression by suppressing the activation of the MAPK (mitogen-activated protein kinase) pathway, which could induce the epithelial–mesenchymal transition and upregulate cyclin-dependent kinase 2 (CDK2) and MMP2 (matrix metallopeptidase 2) expression to promote cell proliferation and metastasis. Therefore, these data suggest that cancer-associated fibroblasts-mediated HCC tumor progression is partially related to the loss of antitumor miR-320a in the exosomes and transfer of stromal cell-derived miR-320a might be a potential treatment option to inhibit HCC progression (96). Exosomes from HCC patient serum contained significantly less HMGN1 (high mobility group nucleosome-binding protein 1) than those from healthy individuals. Exosomes from HCC patient serum contained significantly less HMGN1 (high mobility group nucleosome-binding protein 1) than those from healthy individuals, and the functional domain of HMGN1 is N1ND. Researchers successfully loaded the functional short peptide-N1ND of HMGN1 on the surface of exosomes from HCC, and these treated exosomes efficiently transported N1ND molecules to dendritic cells (DCs) to enhance their activation and immunogenicity. These data demonstrate that N1ND can augment human DC immunogenicity *in vitro* in the presence of tumor-specific antigen and thus provide an avenue for improving DC anti-HCC ability (97). Exosomal EIF3C enhance the angiogenesis and tumorigenesis of HCC, so by inhibiting EIF3C expression could be a potential treatment (66). *In vivo* study exosomal SENP3-EIF4A1 transferred to HCC cells to increase the expression of ZFP36 by competitively binding to miR-9-5p and was capable of inhibiting tumor growth. Thus, exosomal SENP3-EIF4A1 could be a potential therapeutic factor for HCC (85). Cargo sorting of exosomes depends on the endosomal sorting complex required for transport (ESCRT) machinery. Membrane neck cleavage mediated by ESCRT-III is crucial for the formation of exosomes, which is regulated by Vps4, Vps4A and Vps4B. Vps4A is frequently down-regulated in HCC tissues. Vps4A interferes with the biological activity of exosomes and the cell response to exosomes by affecting exosomal miRNA secretion and uptake. Overexpression of Vps4A inactivated phosphatidylinositol-3-kinase (PI3K)/Akt signaling pathway through a comprehensive coordinated effect of the associated miRNAs. These results implicate Vps4A as a novel tumor suppressor for HCC (98). Wang et al. found that miR-1290 is overexpressed in HCC and promotes tumor angiogenesis *via* exosomal secretion. And inhibiting the expression of miR-1290 *in vivo* effectively reduced the tumor angiogenesis and progression, providing evidence that targeting miR-1290 or inhibit the transfer *via* exosome might be a potential strategy for angiogenesis-based cancer therapy (46). Exosomes containing high level of linc-FAM138B (Exo-FAM138B) inhibited HCC growth by modulating miR-765 (49). Exosomes have advantages as ideal drug carriers, and these advantages include but not limited to: 1) easy escape from the host immune surveillance; 2) easy absorption; 3) long circulating half-life, and 4) with the ability of directional homing. So far, exosomes have been used successfully to carry specific molecules, like anticancer genes, inflammatory regulatory factors and other drug resistance-reversing molecules, facilitating personalized treatment of HCC (**Table 2**).

**Table 2:** Exosomes as vehicle for the delivery of therapeutic agents.

Origin of exosomes	Cargo	Outcome	Ref.
BM-MSCs	siGRP78	enhance sensitivity to Sorafenib	(90)
AMSCs	miR-122	increase chemosensitivity	(93)
AMSCs	miR-199a-3p	enhance sensitivity to doxorubic	(94)
HucMSCs	miR-451a	represses epithelial-mesenchymal transition	(95)
HCC-associated fibroblasts	miR-320a	inhibit tumourigenesis	(96)
Tumor cells of HCC	N1ND	argument DC anti-HCC ability	(97)
HCC cells	EIF3C	enhance tumor progression	(66)
HCC cells	Vps4A	repress tumor progression and metastasis	(98)
HCC cells	miR-1290	promote tumor angiogenesis	(46)
HCC cells	linc-FAM138B	inhibited tumor progression	(49)
HuH7 and HL-7702 cells	SENP3-EIF4A1	inhibit tumor growth	(85)

## Conclusions and Future Perspectives

Exosomes have been involved many aspects of HCC, such as occurrence, progression, metastasis and drug resistance. Firstly, exosomes can form microenvironment at local and distant metastasis sites to benefit tumor cell proliferation. Secondly, exosomes secreted by tumor cells can promote angiogenesis in tumor tissue. Thirdly, exosomes released by tumors may play an important role in immune evasion of tumor cells. Finally, exosomes from tumor cells can desensitize the cells to anti-tumor treatment confering drug resistance.

Remarkable progress in exosome research has been made in the past years. The collection and analysis of exosomes released from tumors have become an important research direction for liquid biopsy. Novel therapeutic strategies targeting exosomes may improve the treatment outcomes of cancer patients. However, the mechanisms through which how exosomes regulate tumor development and prognosis need to be further clarified in order to facilitate the development of targeted drugs to better inhibit tumor invasion and growth. For example, EMT has been proposed to be a vital mechanism for epithelial cells to acquire a malignant phenotype. Recently, the role of exosomes in the EMT program has been revealed in different types of cancer, including colorectal cancer and breast cancer, etc (99, 100). However, the underlying mechanisms of exosomes in promoting EMT in HCC cells remain elusive. Owing to difficulties in detecting early HCC in clinical practice, identification of highly specific diagnostic markers, such as exosomes, is urgently required. As the extraction and detection technology of nucleic acid molecules is relatively mature, the research of exosome markers mainly focuses on nucleic acid molecules. The research of exosome protein markers requires high quality exosome separation. Exosome proteins extracted often carry contaminated proteins, and the abundance of these proteins is relatively low, making it difficult to detect. However, the rapid development of proteomics technology will greatly facilitate the study of exosome protein markers.

## Author Contributions

SL produced initial drafts of the manuscript, tables, and figures. LC conceived the idea and edited the manuscript, figures and table drafts. All authors contributed to the article and approved the submitted version.

## Funding

This research was partially supported by the National Key Research and Development Program of China (2018YFE0107500), and the Science and Technology Partnership Program, Ministry of Science and Technology of China (KY201904011).

## Conflict of Interest

The authors declare that the research was conducted in the absence of any commercial or financial relationships that could be construed as a potential conflict of interest.

## Publisher’s Note

All claims expressed in this article are solely those of the authors and do not necessarily represent those of their affiliated organizations, or those of the publisher, the editors and the reviewers. Any product that may be evaluated in this article, or claim that may be made by its manufacturer, is not guaranteed or endorsed by the publisher.
